# Fibronectin-Integrin Signaling Is Required for L-Glutamine’s Protection against Gut Injury

**DOI:** 10.1371/journal.pone.0050185

**Published:** 2012-11-20

**Authors:** Stefanie Niederlechner, Jelena Klawitter, Christine Baird, Alyssa R. Kallweit, Uwe Christians, Paul E. Wischmeyer

**Affiliations:** Department of Anesthesiology, University of Colorado, Aurora, Colorado, United States of America; Seoul National University, Republic of Korea

## Abstract

**Background:**

Extracellular matrix (ECM) stabilization and fibronectin (FN)-Integrin signaling can mediate cellular protection. L-glutamine (GLN) is known to prevent apoptosis after injury. However, it is currently unknown if ECM stabilization and FN-Integrin osmosensing pathways are related to GLN’s cell protective mechanism in the intestine.

**Methods:**

IEC-6 cells were treated with GLN with or without FN siRNA, integrin inhibitor GRGDSP, control peptide GRGESP or ERK1/2 inhibitors PD98059 and UO126 under basal and stressed conditions. Cell survival measured via MTS assay. Phosphorylated and/or total levels of cleaved caspase-3, cleaved PARP, Bax, Bcl-2, heat shock proteins (HSPs), ERK1/2 and transcription factor HSF-1 assessed via Western blotting. Cell size and F-actin morphology quantified by confocal fluorescence microscopy and intracellular GLN concentration by LC-MS/MS.

**Results:**

GLN’s prevention of FN degradation after hyperthermia attenuated apoptosis. Additionally, inhibition of FN-Integrin interaction by GRGDSP and ERK1/2 kinase inhibition by PD98059 inhibited GLN’s protective effect. GRGDSP attenuated GLN-mediated increases in ERK1/2 phosphorylation and HSF-1 levels. PD98059 and GRGDSP also decreased HSP levels after GLN treatment. Finally, GRGDSP attenuated GLN-mediated increases in cell area size and disrupted F-actin assembly, but had no effect on intracellular GLN concentrations.

**Conclusion:**

Taken together, this data suggests that prevention of FN degradation and the FN-Integrin signaling play a key role in GLN-mediated cellular protection. GLN’s signaling via the FN-Integrin pathway is associated with HSP induction via ERK1/2 and HSF-1 activation leading to reduced apoptosis after gut injury.

## Introduction

The small intestinal epithelium undergoes continuous self-renewal, consisting of intestinal cell proliferation, differentiation, and apoptosis. This process is regulated by multiple factors such as luminal nutrients and growth factors [1], [2]. Injuries such as heat stress (HS) result in significant intestinal injury, e.g. heating rats to 41.5–42°C was found to induce a marked increase in intestinal epithelial damage and permeability [3], [4]. In other forms of intestinal injury (i.e. Crohn’s disease and ulcerative colitis), the bowel wall is damaged, which is followed by subsequent inflammation, requiring efficient wound healing for resolution. The production and stabilization of the extracellular matrix (ECM) serves an important role in maintaining the gut barrier and potentially in mediating key cell protection pathway signaling. In the intestinal mucosa, cell stress (such as heat injury and inflammation) is followed by degradation of ECM and loss of epithelial cells, leading to loss of gut barrier function and ulceration [5]. New therapeutics, aimed at preventing tissue damage and enhancing ECM synthesis, could be of benefit in clinical states of intestinal injury [5].

ECM proteins such as fibronectin (FN), laminin, and collagen have been shown to play a critical role in tissue repair and survival in intestinal injury [6]. FN, a high molecular weight adhesive glycoprotein in the basement membrane and connective tissue matrices of the intestine is known to be vital to cell adhesion, migration, growth, and wound repair. These effects are dependent on binding of FN to the integrin receptor [6]. FN expression is important for the maintainance of normal epithelial integrity as well as for the regulation of epithelial response to intestinal injury [7]. FN contains an Arg-Gly-Asp (RGD) attachment site, which can bind to integrin receptors forming an important recognition system for cell adhesion and survival signaling pathways [8], [9].

Integrins are cell surface transmembrane heterodimeric glycoproteins, which establish cell adhesion to the ECM [10], [11]. This pathway of integrin-mediated cell adhesion has been found to play a critical role in the control of cellular apoptosis [9].

Cells bind and exert forces on fibronectin through integrins, which mechanically couple the actin cytoskeleton to the ECM via an elaborate adhesion complex. This adhesion complex mediates cell matrix adhesion, but also serves as a surface anchor for the cytoskeleton. Therefore, it is able to transduce signals from the ECM to the cell and vice versa [10]. F-actin assembly plays an important role in inflammation, cell size regulation and apoptosis [12], [13], and is regulated via FN-Integrin signaling [14].

Protein tyrosine phosphorylation is a significant biochemical event for integrin-dependent functional responses [10]. It has been shown that the p44/p42MAPK (ERK1/2) signaling pathway is stimulated by growth factors and adhesion signals from integrins [15].

L-glutamine (GLN), known to be an osmotically acting amino acid, can be cytoprotective following injury *in vitro* and *in vivo* by reducing bacterial translocation, protecting the gut mucosa against injury, and modulating immune function [16], [17], [18], [19], [20], [21]. GLN is felt to be a conditionally essential amino acid, as it is mobilized to the circulation from muscle stores in large amounts following stress and injury [22]. GLN is primarily co-transported into cells by Na^+^-dependent systems, which cause cell swelling because of the influx of water [23]. Cell swelling mechanisms have been associated with the regulation of metabolism, activation of transcription factors, and the regulation of gene expression, such as heat shock proteins (HSP), following cellular stress [24], [25], [26], [27]. However, it is currently unknown if GLN-mediated cell swelling modulates gene expression in states of cellular stress and injury. Further, the precise mechanism of GLN-mediated cellular protection remains unknown.

Extensive studies were performed in Haussinger’s and Schliess research groups concerning cell volume changes and cell survival. They showed that cell shrinkage supports a catabolic situation and that cell swelling exerts growth factor-like effects, by activating ERK1/2 [28].

Our laboratory has previously shown that following cellular injury GLN enhances transactivation of transcription factor HSF-1, which activates key protective stress response pathways, such as enhanced HSP70 expression [29], [30], [31]. Further, GLN can decrease caspase-3 and poly (ADP-ribose) polymerase (PARP) cleavage after HS, leading to a significant reduction of cellular apoptosis [32]. As previously described, the integrin cell volume-sensing pathway has also been shown to be a key protective pathway following stress [33], [34].

Given this existing data, we hypothesized that the mobilization of GLN into the cirulcation following stress my serve as a “cytoprotective signal” leading to GLN-mediated cell swelling and modulation of FN-Integrin-mediated signaling. We further hypothesize that this activation of FN-Integrin signaling modulates GLN’s activation of key stress response proteins, such as HSP70, via ERK1/2. Therefore, this study specifically investigates the role of prevention of FN degradation and FN-Integrin signaling via ERK1/2, HSF-1, and HSP in GLN’s molecular mechanism of cellular protection.

## Materials and Methods

### Chemicals

All chemicals were purchased from Sigma-Aldrich (St. Louis, MO), unless otherwise specified.

### Cell Culture

IEC-6 cells (ATCC, Manassas, VA) were cultured as previously described [35].

### Heat-stress Injury

For cell viability, 96-well plates were submerged to a lethal HS in a 44°C Precision water bath Model 260 (Winchester, VI) for 50 min [35] and allowed to recover at 37°C for 24 h. For protein expression, microscopy and LC-MS/MS experiments, cells were subjected to a non-lethal HS at 43°C for 45 min [35] and were allowed to recover for either 0 min for measurement of phosphorylated proteins, FN expression and transfection, 15 min for microscopy and LC-MS/MS experiments, or 3 h for FN, HSP, total HSF-1, caspase-3 and PARP expression.

### Small Interfering (si) RNA Transfection

Cells were seeded in 10-cm dishes and allowed to grow for 24 h (50–60% confluence) in full media. Media was changed to DMEM (with 2 mM GLN) plus 10% FBS only, and cells were transfected for 48 h using SilentFect (Bio-Rad, Hercules, CA) with either no siRNA, FN siRNA (20 nM), or control noncoding (NC) oligos (20 nM) with a comparable GC content to the FN siRNA (Invitrogen). Cells were starved with DMEM (containing 0 mM GLN) plus 10% FBS only 24 h before HS (transfection reagents still present). Cells were then treated with 0 or 10 mM GLN and subjected to HS, as described above.

### Western Blot Analysis

Cells were seeded in 10 cm dishes and allowed to grow for 3 d in full media, before the cells were starved in GLN-free, serum containing DMEM for 24 h. Cells were then treated with and without 10 mM GLN for 15 min, with or without prior 1 h treatment with FN-Integrin inhibitor GRGDSP (50 µM), its negative control peptide GRGESP (50 µM) (Bachem, Torrance, CA) or ERK1/2 kinase inhibitor PD98059 (50 µM) (Calbiochem, Philadelphia, PA), and subjected to HS (as above). At the end of experimental treatment, Western blotting was performed as previously described [35]. Primary antibodies against HSP32 and HSP70 (1∶10,000 dilution) (StressGen, Victoria, BC, Canada) and FN (1∶10,000), or against total ERK1/2, [T(P)^202^/Y(P)^204^]ERK1/2, total HSF-1, [S(P)^303^]HSF-1, cleaved caspase-3, cleaved PARP, bax, and bcl-2 (1∶1,000) (Cell signaling, Danvers, MA) were used.

### Cell Viability

IEC-6 cells were seeded in 96 well plates (7,000 cells per well), and allowed to grow for 42 h in full media until 80% confluence. The cells were, then, cultured for 24 h in GLN-free, serum containing DMEM. After GLN-starvation for 24 h, cells were exposed to different concentrations of GLN (0, 2, 10, and 20 mM) for 15 min. 50 µM GRGDSP, 50 µM PD98059, 30 µM UO126 (Cell signaling, Danvers, MA), or 50 µM GRGESP were used 1 h prior to GLN treatment. Cells were, then, subjected to lethal HS (as specified above) and cell viability was evaluated 24 h post HS as previously described [35]. All groups were normalized to their non-HS controls to account for differences in cell proliferation.

### Confocal Fluorescence Microscopy

Starved IEC-6 cells were treated with 0, 2 or 10 mM GLN for 15 min with or without subsequent non-lethal HS injury (as above). Specified groups were pretreated with GRGDSP or GRGESP (50 µM) for 1 h prior to GLN treatment. Confocal fluorescence microscopy was performed as described [35]. Slides were examined 15 min post-HS for cell area size and F-actin morphology, using a Zeiss Axiovert inverted microscope (Carl Zeiss Inc, Thornhood, NY). A minimum of 50 cells was evaluated for each group in each experiment. Data was collected using Slidebook 3.0 software (Intelligent Imaging Innovations Inc., Denver, CO).

### Measurement of GLN in Cell Extracts using Tandem Mass Spectrometry (LC-MS/MS)

Cells were seeded in 10 cm dishes and allowed to grow for 3 d in full media, before the media was replaced with GLN-free, serum containing DMEM for 24 h. Starved cells were treated with and without 10 mM GLN for 15 min, with or without prior 1 h treatment with 50 µM GRGDSP or 50 µM GRGESP, and subjected to HS (as above). For quantitation of amino acids in cell extracts, cell monolayers were washed five times with ice-cold PBS, scraped into PBS (1000 µL), and centrifuged (14,000 rpm, 10 min) to remove cell debris. PBS was discarded, acetonitrile/methanol mix was added, snap frozen two times, and sonicated on ice for 5 minutes.

An API4000 triple quadruple MS system (Applied Biosystems Inc, Framingham, MA) coupled to an Agilent 1100 HPLC (Santa Clara, CA) and a Leap CTC Autosampler (Carrboro, NC) was used for the LC-MS/MS analysis of GLN and Glutamate concentrations. In order to avoid amino acid derivatization and preventing interferences between related amino acids (glutamine/glutamate), the amino acids were baseline HPLC separated on a Synergi Polar 4u column (250×3 mm, 4 µm particle size, Phenomenex, Torrance, CA). The LC mobile phases consisted of 5 mM ammonium acetate supplemented with 0.05% trifluoric acid (TFA) in water (mobile A) and methanol (mobile B). The following LC gradient was run: 0–0.8 min: 1%B; 0.8–3 min: 1–6%B; 3–5 min: 6–50%B; 5–6 min: 1%B. MS data was acquired in negative electrospray ionization mode with an ion voltage of −4.2 kV. Stable-isotope labeled glutamine (U-^13^C5-glutamine; Cambridge Isotope, Inc., Andover, MA) was used as internal standard for the calibration and normalization. The MS MRM (multiple reaction monitoring) transitions were as follows: glutamine: 145.1→126.9, glutamate: 146.1→127.8/101.9, and U-^13^C5-glutamine: 149.8→113.9. Data was acquired and processed for calibration and quantification of all analytes using the Analyst software 1.4.2 (SpectraLab Scientific Inc., Alexandria, VA).

Compound stock solutions, calibration standards and quality controls were prepared in acetonitrile/water mixture (10/90 v/v). Samples were diluted 1∶10 (v/v) with the acetonitrile/water (10/90) solvent mixture containing the internal standard (1 µM). After centrifugation, diluted samples (20 µL) were injected and analyzed by LC-MS/MS. Samples were normalized to their cell number counts, measured by using a Nexcelom Cellometer™ Auto T4 from BD Bioscience (San Jose, CA).

### Data Analysis and Statistics

All experiments were repeated at least 3 times with IEC-6 cells of different passage numbers (15–20). Statistical analysis was validated with GraphPad Prism Analysis software. Conditions were compared by two-tailed student’s *t*-test and data are given as means±SEM (number of experiments). Differences were considered significant at *P*<.05.

## Results

### GLN-mediated Cellular Protection via Prevention of FN Degradation after HS

**Figure 1:**
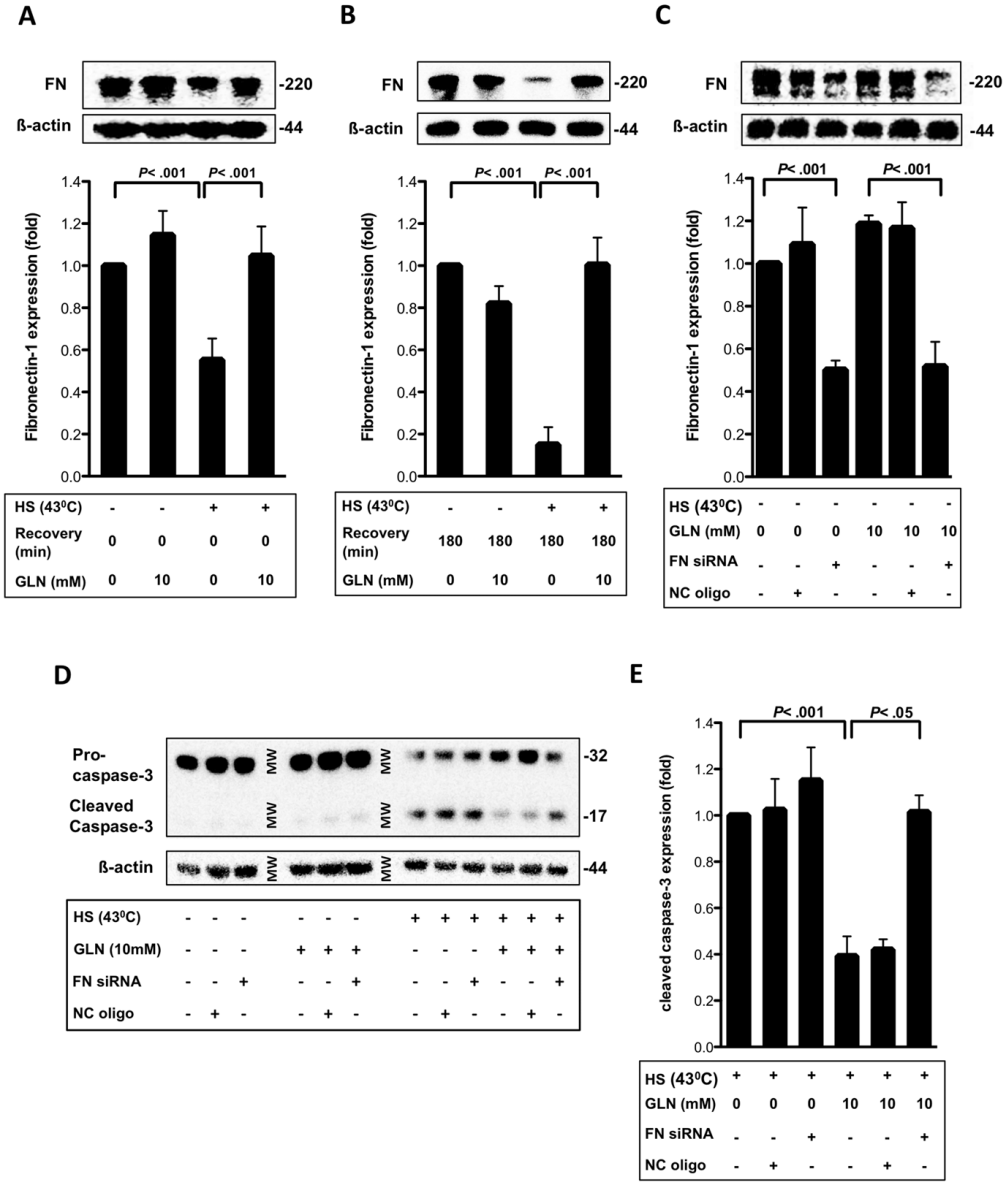
GLN is protective by preventing FN degradation after HS. A) FN levels from IEC-6 cells, with or without GLN treatment under unstressed or stressed conditions, were determined by Western blot with ß-actin as loading control after 0 h recovery. Densitometric analysis of FN expression as mean fold change relative to 0 mM GLN cells±SEM (n = 4). B) Western blot and densitometric analysis of FN after 3 h recovery is shown (n = 4). C) FN expression of transfected IEC-6 cells with no siRNA, NC siRNA, or FN siRNA (n = 3) are presented. D) Caspase-3 and cleaved caspase-3 expression was determined via Western blot in basal and HS conditions after IEC-6 cells were transfected with no siRNA, NC siRNA, or FN siRNA (n = 3). E) Densitometric analysis of cleaved caspase-3 levels is shown as mean fold change relative to HS 0 mM GLN cells ±SEM (n = 3).

FN expression was significantly reduced by 50% in IEC-6 cells immediately following HS and by 85% at 3 h post-HS (*P*<.001). GLN treatment (10 mM) prevented this decrease in FN expression after HS (*P*<.001) (Fig. 1A and 1B). GLN-mediated preservation of FN expression was associated with a reduction of cleaved caspase-3 levels, indicating a reduction in cellular apoptosis (Fig. 1D and 1E). To demonstrate that FN expression is important in GLN-mediated protection of IEC-6 cells, cells were transfected with 20 nM FN siRNA. Fig. 1C shows a 50% decrease in FN expression after FN siRNA treatment (*P*<.001). Attenuation of FN expression via siRNA led to a significant decrease of GLN-mediated protection shown by increases of cleaved caspase-3 levels following HS (*P*<.001) (Fig. 1D and 1E). This indicates GLN-mediated protection against apoptosis after HS was lost when FN expression was attenuated. As expected, we typically observed multiple bands scattered across 200–250 kDa, the expected molecular weight range for fibronectin fragments [36]. It is interesting to note these multiple bands appear to be lost following HS. GLN-treatment in HS cells appears to preserve this double or multiple band appearance (Figure 1A–1C). As integrin fragments have been hypothesized to play a key role in integrin binding and cellular cell adhesion, migration, and repair, the apparent loss of these fragments following HS may be an important contributor to the disrupted ECM interactions following stress that may contribute to impaired cell repair [37].

### Involvement of FN-Integrin Interaction in GLN-mediated Cellular Protection

**Figure 2:**
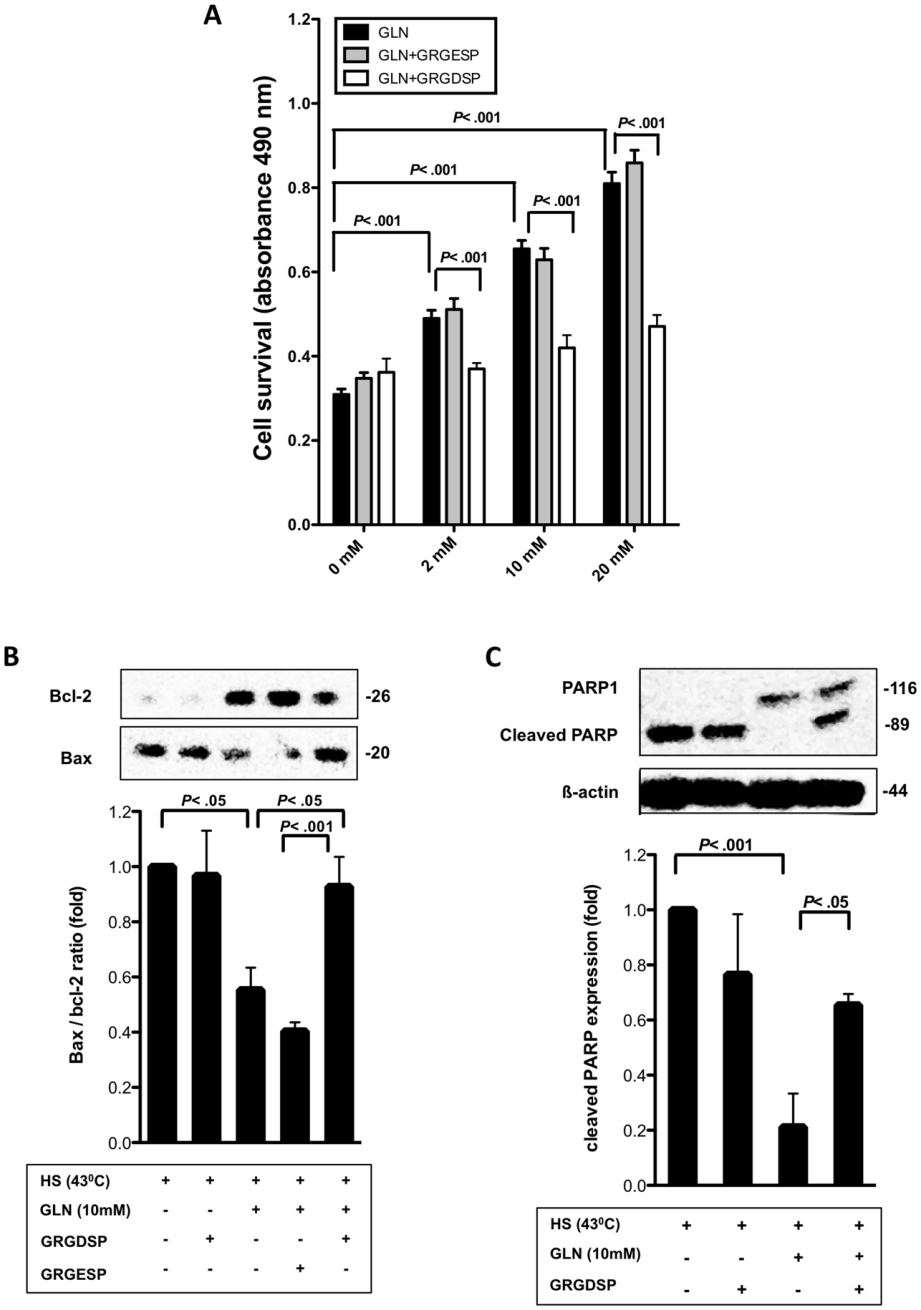
GLN’s intestinal protection is inhibited by GRGDSP. A) IEC-6 cells were treated for 1 h with either media, GRGDSP or GRGESP before the cells were treated with 0, 2, 10 or 20 mM GLN. Cell survival, following lethal HS (44°C), was measured via MTS assay. All groups were normalized to their non-HS controls to account for differences in cell growth. Assays were carried out in triplicate, experiments were performed 4 times and shown as mean±SEM. B) IEC-6 cells were treated as described in Fig. 2A, but they underwent non-lethal HS (43°C). After 3 hours recovery at 37°C, Bax and Bcl-2 levels were measured by Western blot. Bax was ratioed to anti-apoptotic marker Bcl-2 and shown in fold change±SEM (n = 4). C) Representative Western blot of PARP and cleaved PARP and densitometric analysis of cleaved PARP ratioed to HS 0 mM GLN are displayed. Cleaved PARP levels are presented as fold change±SEM (n = 4).

The effect of the FN-Integrin interaction inhibitor GRGDSP on GLN-mediated cellular protection following lethal HS was evaluated via MTS assay (Fig. 2A). Control groups were added to measure any possible toxicity of the inhibitor reagents. As shown in Fig. 2A, GRGDSP alone did not affect cell viability. GLN increased cell survival in HS cells in a dose dependent manner (*P*<.001). Importantly, GRGDSP completely attenuated GLN’s protection at a concentration of 2 mM GLN (*P*<.001). At higher GLN concentrations (10 mM–20 mM), GRGDSP decreased GLN’s protective effect by ∼ 85% (*P*<.001). The control peptide GRGESP had no effect on GLN`s protective effect in either group (Fig. 2A).

MTS cell survival assays measure mitochondrial dehydrogenases activities, which may be preserved in cells with compromised barriers. In addition, key apoptotic pathway makers were evaluated via Western blotting, confirming the MTS cell survival results. HS increased apoptotic markers such as Bax/bcl-2 ratio and cleaved PARP levels. GLN supplementation attenuated markers of apoptosis after HS, indicating that GLN can improve cell survival and prevent apoptosis during HS (Fig. 2B–C). Specifically, GLN decreased Bax/bcl-2 ratio levels by 45% (*P*<.05) and cleaved PARP levels by 79% (*P*<.001). However, GRGDSP treatment decreased GLN-mediated anti-apopototic effects as shown by increased Bax/bcl-2 ratio (*P*<.001) and PARP levels (*P*<.05) (Fig. 2B–C). This data suggests that in the absence of FN-Integrin signaling, GLN’s cellular protection against hyperthermia is lost.

### GRGDSP Inhibits GLN-mediated Increases in Cell Area Size, Disrupts F-actin Organization, but has no Impact on Intracellular GLN Concentrations

**Figure 3:**
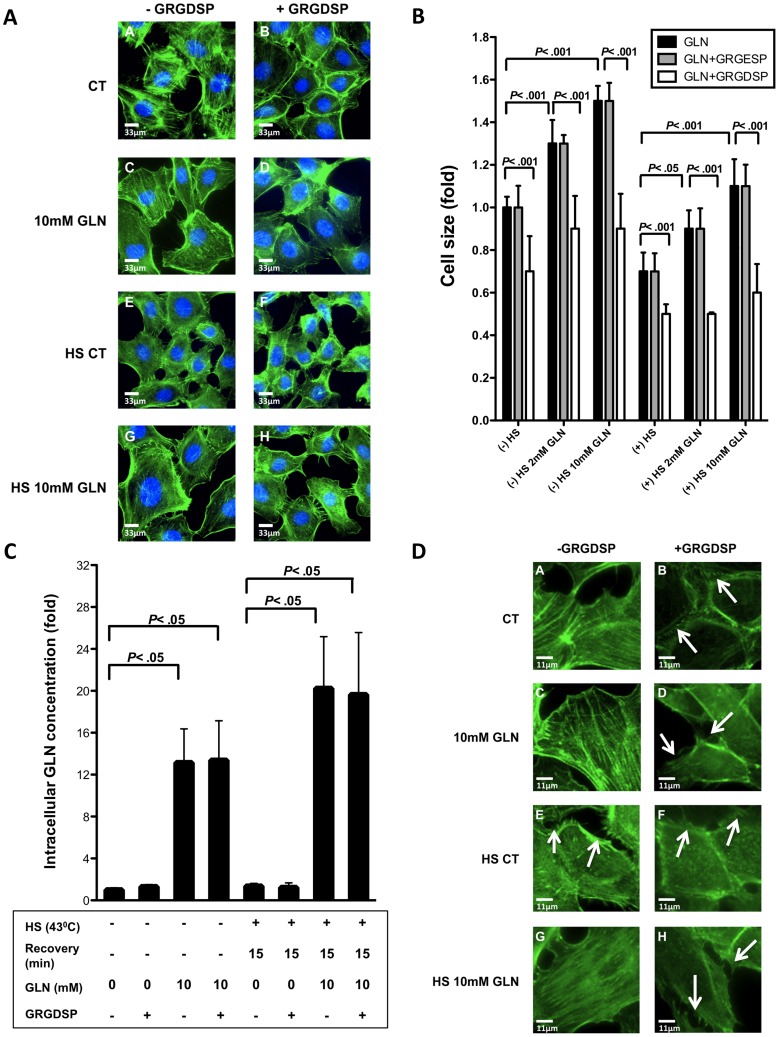
GRGDSP’s effect on cell area size, F-actin morphology and intracellular GLN concentration. A) Cell area size is shown by fluorescence microscopy in IEC-6 cells under 0 mM GLN conditions and 10 mM GLN treatment without inhibitor GRGDSP (Images A, C, E, G) or after 1 h prior treatment with GRGDSP (Images C, D, F, H) in basal and stressed conditions (43°C). F-actin is shown in green and nuclei in blue. All images taken 15 min following exposure of cells to GLN in non-HS cells and 15 min post-HS in stressed cells. All images were aquired using the same exposure times, and were renormalized the same for optical comparison (n = 4). Scalebars = 33 µm. B) Cell area size data are shown in fold increase under non-HS (37°C) and HS (43°C) conditions (n = 3–4) C) IEC-6 cells were treated as descibed in A). GLN levels in cell extracts were quantified using LC-MS/MS and are shown as fold change± SEM ratioed to 0 mM GLN group (n = 6–7). D) Fluorescence microscopy was used for the morphological analysis of the distribution of F-actin. Microfilaments were visualized with Alexa Flour 488 phalloidin antibody. Representative results are shown from 4 independent fluorescence microscopy experiments for each condition. Scalebars: 45 µm.

Fluorescence microscopy was utilized to visualize GLN-mediated cell swelling in the presence and absence of GRGDSP. We analyzed the cell area size of a large number of cells to attempt to maximize accuracy of findings (50 cells per group in each of the 3–4 experiments). Each cell was masked using Slidebook 3.0 software and total cell area size statistics were exported into GraphPad Prism 5.0 b. Based on these cell area measurements, we were able to confirm that GLN treatment (2 mM and 10 mM) caused cell swelling, which was inhibited after GRGDSP treatment in a robust fashion in IEC-6 cells. GLN increased cell area size in non-HS cells by 1.3 fold in the 2 mM GLN group and 1.5 fold in the 10 mM GLN group (*P*<.001). In HS cells, GLN increased cell area by 1.4 fold in the 2 mM GLN group (*P*<.05) and 1.7 fold in the 10 mM GLN group (*P*<.001). Treatment with GRGDSP fully inhibited GLN-mediated cell swelling in all groups with a slight decrease in cell area size observed in the CT groups (*P*<.001). Control peptide GRGESP had no effect on GLN-mediated cell area size changes (Fig. 3A and 3B).

As reduced intracellular GLN leads to cell shrinkage [23], we next investigated if GRGDSP treatment led to decreased intracellular GLN concentrations in basal and stressed conditions. LC-MS/MS results, shown in Fig. 3C, demonstrate that GRGDSP treatment had no impact on intracellular GLN content in basal or stressed conditions (*P*<.05).

Cytoskeletal structures, such as F-actin, play an important role in inflammation and in determining cell shape and cell size [12], [13]. To further investigate possible causes of cell-shrinkage following GRGDSP treatment, fluorescent staining of F-actin cytoskeleton by confocal microscopy revealed its’ intracellular disruption after HS and after GRGDSP treatment. In unstressed cells and GLN-treated cells, post-HS F-actin is intact, continuous, and smooth. In contrast, after GRGDSP treatment in 0 mM and 10 mM GLN treated groups, F-actin appears to be fragmented, disrupted, disorganized and collapsed (arrows) (Fig. 3D).

### GLN is Protective by Activating ERK1/2 via the FN-Integrin Pathway

**Figure 4:**
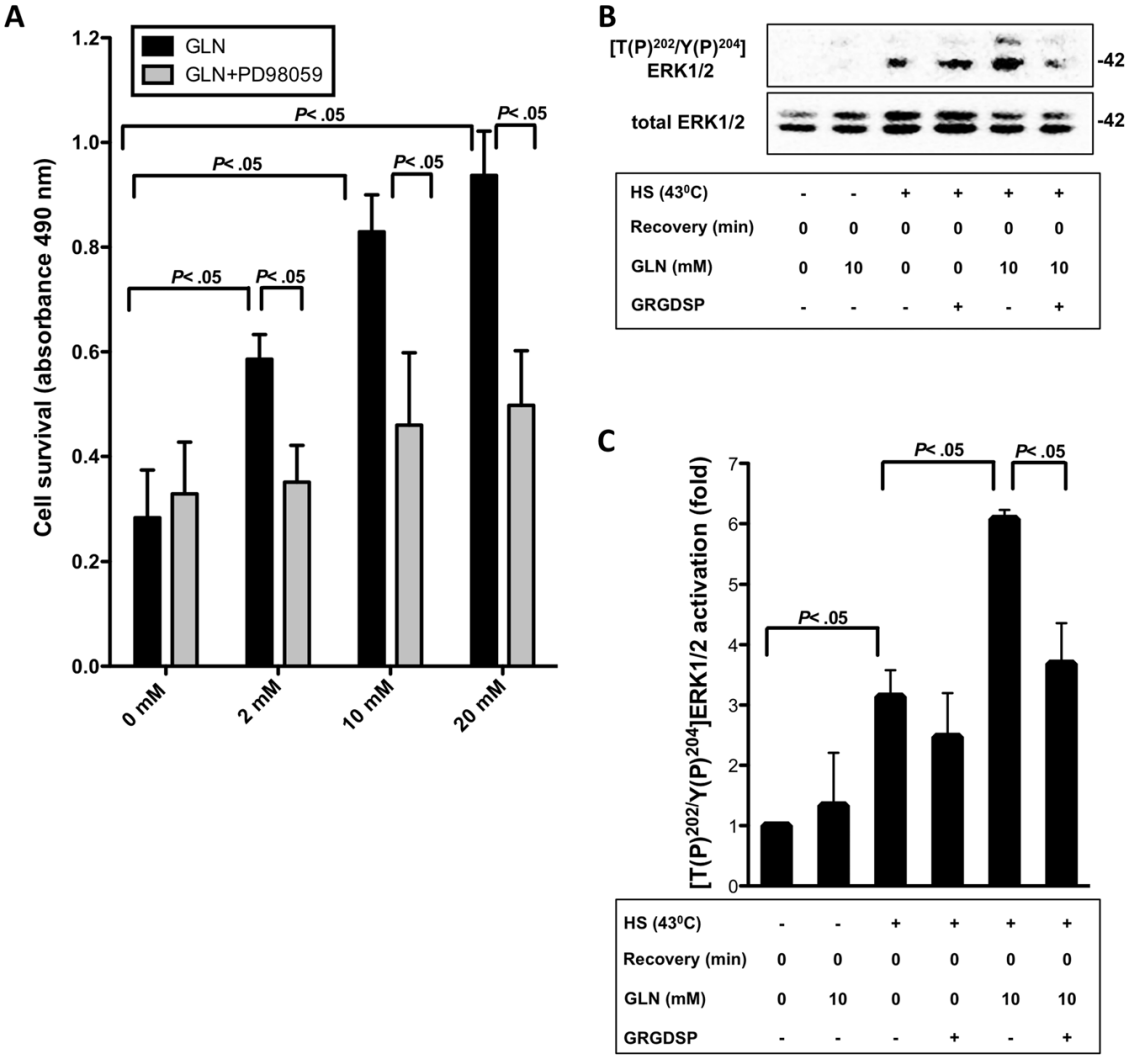
ERK1/2 activation is involved in GLN’s protective mechanism and attenuates after FN-Integrin pathway inhibition. A) IEC-6 cells were treated with different concentrations of GLN (0, 2, 10, and 20 mM) with or without 1 h prior PD98059 treatment. Cell survival was measured via MTS assay. Results are shown as mean±SEM (n = 3). B) [T(P)^202^/Y(P)^204^]ERK1/2 and total ERK1/2 levels were determined by Western blot analysis after basal and stressed 43°C conditions without recovery. ERK1/2 activation is shown as mean fold change relative to total ERK1/2±SEM and ratioed to 0 mM GLN (n = 3).

To explore alternative signaling pathways known to be vital to HSP-mediated survival pathways and affected by integrin signaling [38], we examined GLN’s effect on ERK1/2 activation. By inhibiting the ERK1/2 kinase with PD98059 and UO126, we demonstrated that GLN’s protection was inhibited (*P*<.05) (Fig. 4A and Fig. S1). Furthermore, Western blots of [T(P)^202^/Y(P)^204^]ERK1/2, ratioed to total ERK1/2 showed that HS increased phosphorylated ERK1/2 by 3 fold (*P*<.05). GLN treatment further elevated ERK1/2 phosphorylation by 6 fold after HS (*P*<.05). Importantly, this GLN-mediated increase in ERK1/2 phosphorylation was inhibited after GRGDSP treatment (*P*<.05) (Fig. 4B and 4C).

### Activation and Expression of Transcription Factor HSF-1 is Dependent on the FN-Integrin-ERK Pathway

**Figure 5:**
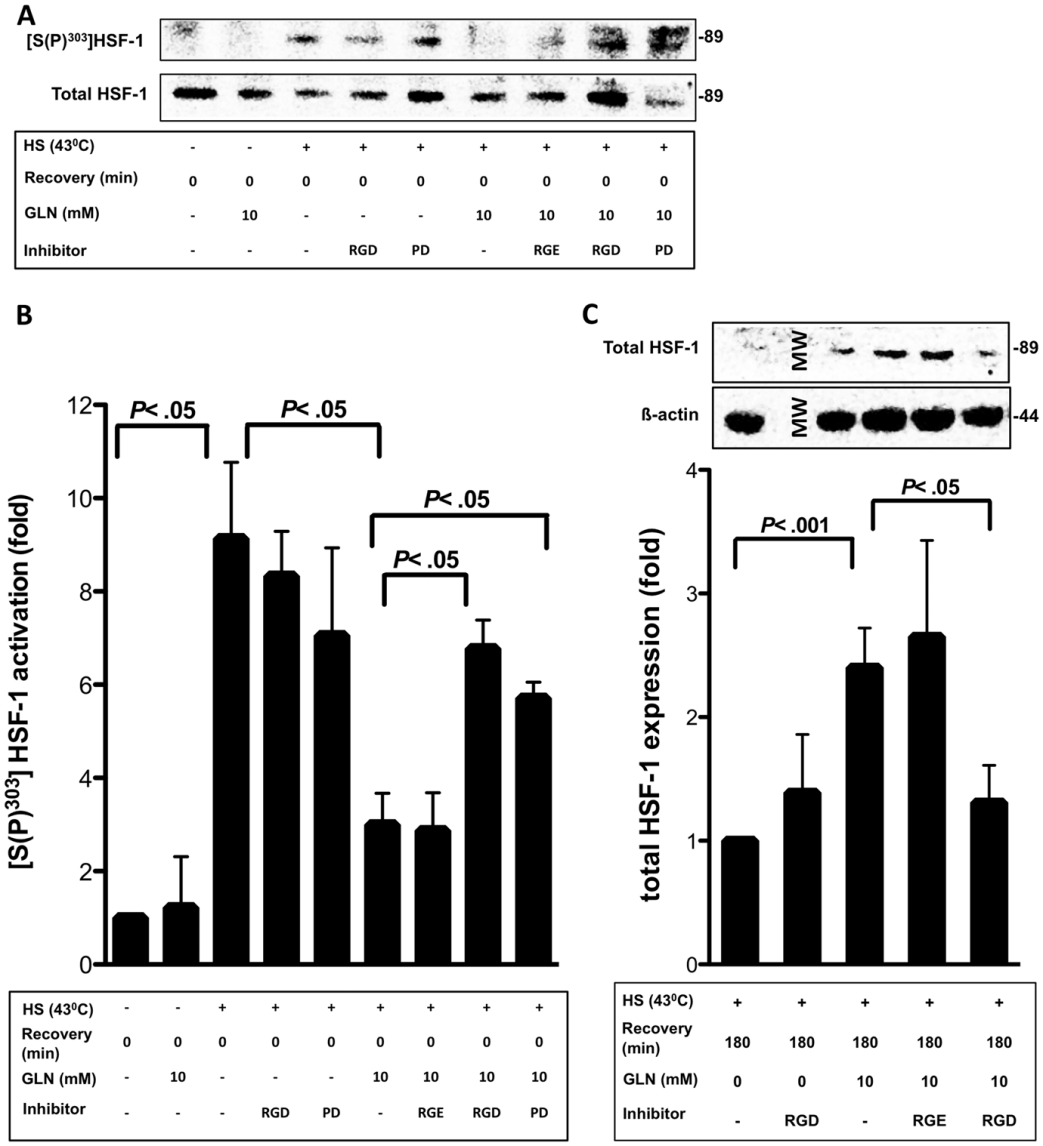
FN-Integrin signaling inhibitor GRGDSP and ERK1/2 kinase inhibitor PD98059 attenuate GLN-mediated activation of HSF-1. A) IEC-6 cells were treated with 0 mM or 10 mM GLN with or without 1 h prior PD98059 or GRGDSP treatment. Representative Western blot from 3 independent experiments shows [S(P)^303^]HSF-1 and total HSF-1 levels. B) Densitometric analysis of HSF-1 phosphorylation at serine 303 was measured as mean fold changes relative to total HSF-1±SEM (n = 3). C) Total HSF-1 levels were measured after 3 h recovery after non-lethal HS. Results are displayed as fold change±SEM ratioed to HS 0 mM GLN groups (n = 3).

Since both ERK and HSF-1 phosphorylation are important in cell survival after HS [39], [40], we investigated if ERK and FN-Integrin signaling played a role in HSF-1 activation and expression. We initially looked at total HSF-1 expression and found that GLN could enhance total HSF-1 expression (*P*<.001). GLN-mediated increases in HSF-1 expression after HS and 3 h post-recovery were attenuated after GRGDSP (*P*<.05), but not by GRGESP (Fig. 5C).

Next, we investigated HSF-1 phosphorylation at phosphorylation site serine 303 after GRGDSP and PD98059 treatment since previous research indicates ERK may participate in the activation of HSF-1, leading to the induction of HSP expression [41]. HSF-1, when phosphorylated at serine 303, is repressed and found as a monomer. Upon activation, HSF-1 forms trimers, gains DNA binding activity and translocates to the nucleus [42]. In non-HS cells and HS cells treated with GLN or GLN+control GRGESP, HSF-1 is not phosphorylated at serine 303. However, in HS cells without GLN, and HS cells treated with GLN+GRGDSP and GLN+PD98059, HSF-1 undergoes inhibitory phosphorylation at serine 303 (*P*<.05) (Fig. 5A and Fig. 5B).

### Inhibition of the FN-Integrin Pathway Attenuates Increases in GLN-mediated HSP Expression after HS

**Figure 6:**
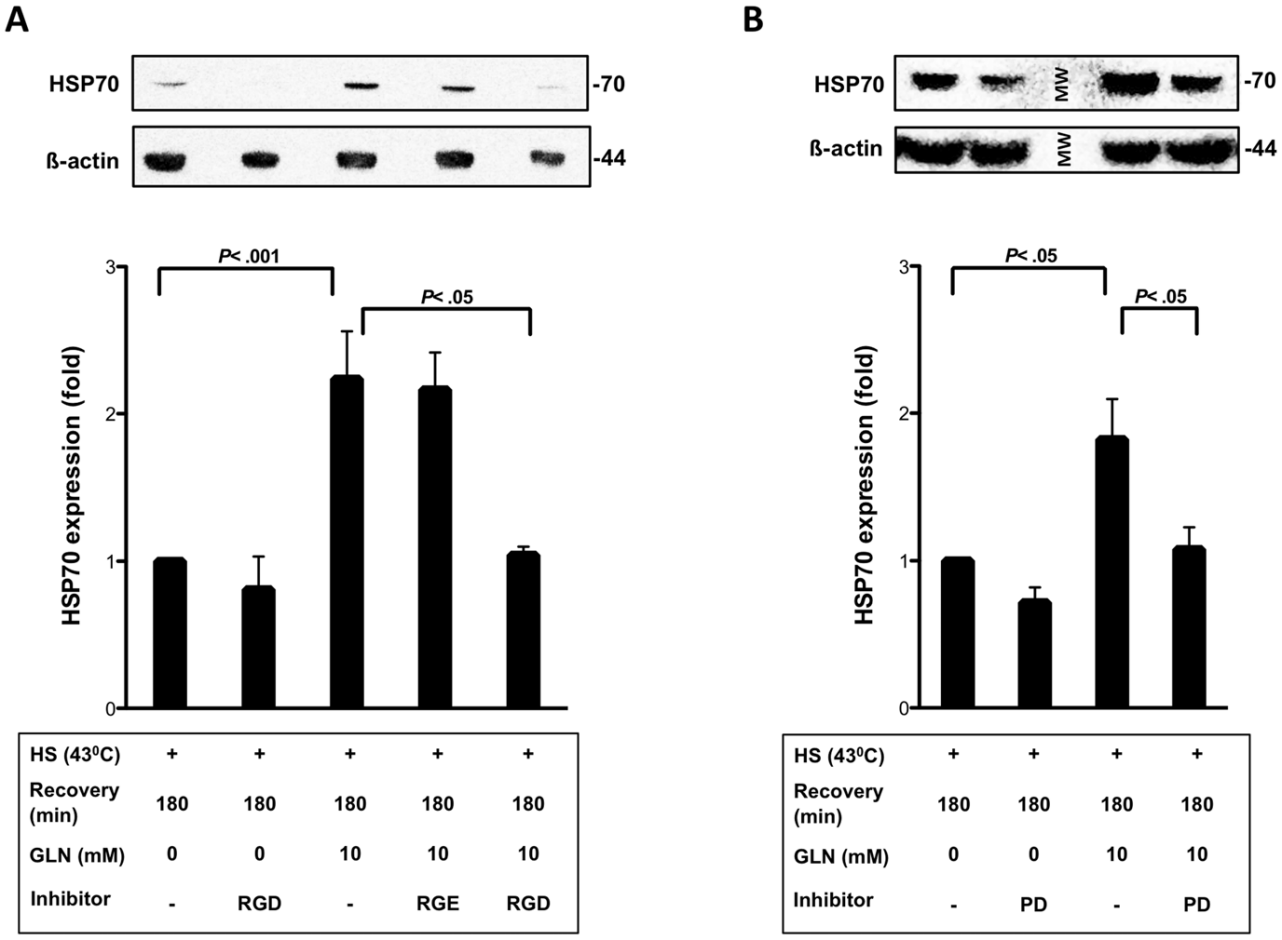
GRGDSP and PD98059 affect GLN-mediated increases in HSP70 expression. A) IEC-6 cells were treated as described in Fig. 2B. HSP70 expression was determined by Western blot analysis. In addition, ß-actin was monitored to normalize total blotted protein. Data are shown as mean fold change relative to HS 0 mM GLN±SEM (n = 5). B) Western blot of HSP70 and ß-actin are shown after PD98059 treatment. Results are ratioed to HS 0 mM GLN groups and represent means±SEM (n = 3).

Enhanced nuclear translocation of HSF-1 is required for augmented HSP70 expression [35]. Our laboratory has shown GLN’s cytoprotective effect is, at least in part, mediated by increased HSP70 expression [31]. To determine the effect of GRGDSP and PD98059 on GLN-mediated HSP70 expression, we examined the expression of HSP70 after HS in IEC-6 cells. As shown in Fig. 6A and 6B, IEC-6 cells treated with GLN significantly increased HSP70 expression after HS (*P*<.05). Cells treated with GRGDSP and PD98059, but not GRGESP, showed a significant decrease in GLN-mediated HSP70 expression (*P*<.05) (Fig. 6A and Fig. 6B).

**Figure 7:**
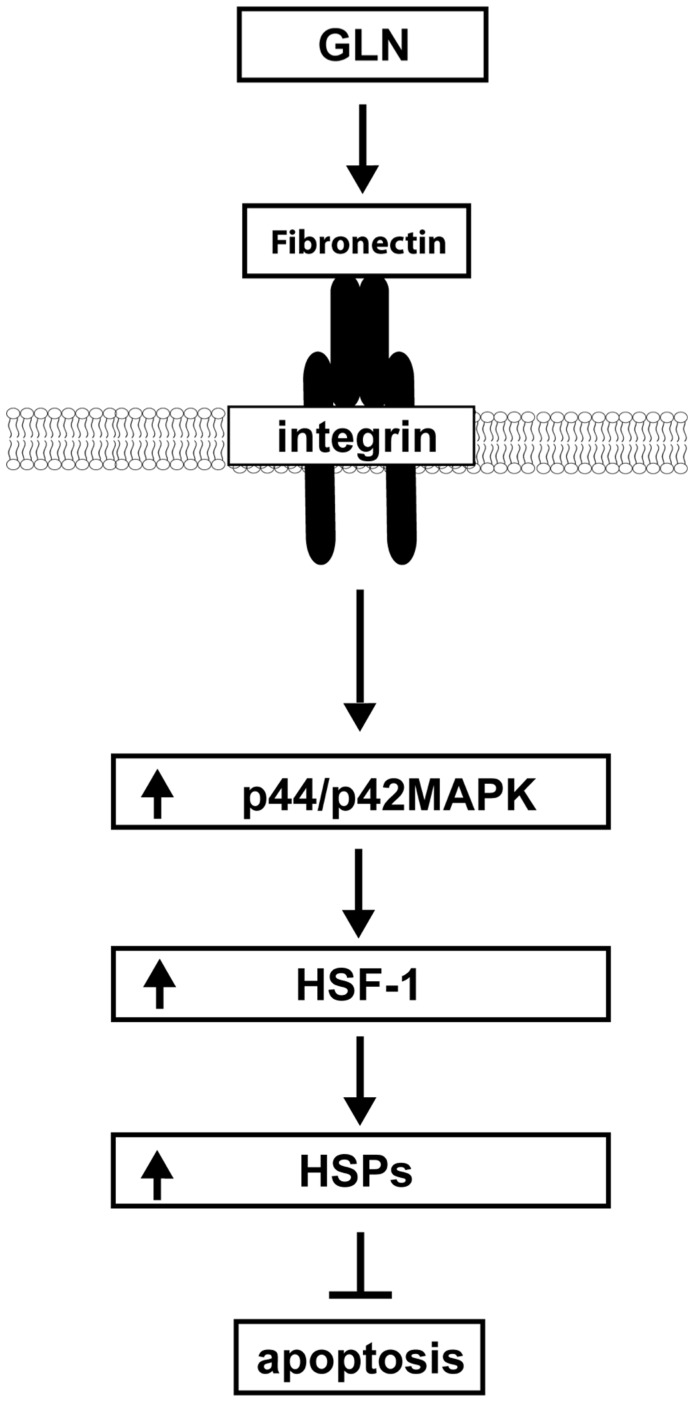
Proposed working model. GLN is protective in the intestine by preventing FN degradation after thermal injury, as well as by activating the protective FN-Integrin signaling pathway. GLN phosphorylates ERK1/2 via the FN-Integrin pathway leading to HSF-1 activation, which enhances HSP expression to prevent apoptosis.

Another recently discovered cytoprotective effect of increased cellular GLN concentration is the induction of HSP32 expression [43]. Here, we show that GLN increased HSP32 expression by 3,5 fold after HS (*P*<.001). GRGDSP, but not GRGESP, decreased this enhancement in HSP32 expression by 70% (*P*<.05) (Fig. S2).

## Discussion

FN is a vital structural component of the basement membranes in the crypts of Lieberkuehn in the gut [44], [45]. The expression of this key basement membrane protein has been shown to be essential to the maintainance of cellular integrity and to the intestinal epithelial cell’s response to injury [7]. Utilizing IEC-6 cells, a small intestinal crypt epithelial cell line, we show that under non-stress conditions intestinal epithelial cells produce substantial amounts of FN. Immediately after HS injury, FN levels were reduced by 50% and by 85% at 3 h post-injury. These reduced levels were associated with significant apoptosis. GLN was able to preserve epithelial cell FN levels following HS to pre-injury levels. The preservation of FN levels seems to correlate with reduced apoptosis (Fig. 1). Here we show for the first time, that GLN can rescue intestinal epithelial cells *in vitro* from cell death by preventing a decrease in FN expression after HS injury (Fig. 1). Silencing FN inhibited GLN’s protection (Fig. 1D and 1E), suggesting that FN expression is essential in GLN’s protective mechanism. Both insufficient and excessive FN expression impair gastrointestinal function and can lead to worsening of disease in patients with Crohn’s disease [5]. Our results demonstrate that GLN prevented the decrease in FN expression and did not appear to lead to excessive expression of FN (Fig. 1A and 1B).

ECM protein stabilization and amino acid-mediated cell hydration changes can modulate many cellular pathways, including cell survival pathways, by initiating signal transduction [44], [46], [47]. Herein, we show not only the prevention of ECM degradation is important in GLN’s protective mechanism after intestinal injury, but also cellular attachment to integrin receptors (Fig. 2,3,4,5,6). To this point, the initiation steps in GLN’s cytoprotective molecular mechanism have remained a mystery. In this study, evidence is presented that integrins may play a key initial role in GLN-induced cell swelling by preserving the anchorage to the ECM. Therefore, we quantified cell area size changes caused by GLN by measuring the cell area size in IEC-6 cells using confocal fluorescence microscopy. We were interested in the possibility of GRGDSP affecting cell area size. GLN caused cell swelling in IEC-6 cells in a dose-dependent manner. However, inhibition of FN-Integrin signaling via GRGDSP fully decreased GLN-mediated cell swelling (Fig. 3A and 3B). One explanation for this attenuation in cell area size may be that after the integrin inhibitor GRGDSP blocks the binding sites for the ECM proteins, signal transduction is reduced which leads to cell shrinkage despite the presence of sufficient GLN. Our data agrees with literature showing that, prior to and during apoptosis, loss of cell volume can be observed [48], [49].

Volume decrease and actin disruption have been shown in previous studies to be related with apoptosis. In this study, we could show that HS and the interruption of the FN-Integrin interaction by GRGDSP lead to cell shrinkage. GLN, however, is able to protect IEC-6 cells by volume increase and actin stabilization via FN-Integrin signaling. If FN-Integrin signaling is interrupted, GLN is unable to be protective via ERK1/2 and HSF-1. Moreover, GLN could prevent heat stress-mediated decreases in FN expression. Less FN may lead to reduced FN-Integrin interaction, following altered FN-Integrin osmosignaling, causing apoptosis. What effect FN degradation has on cell volume, ERK1/2, HSF-1 and HSP70 expression still needs to be investigated in future studies. Here, evidence is presented that its expression plays an essential role in GLN’s protective mechanism to prevent apoptosis shown via cleaved caspase-3 levels (Fig. 1 D–E).

After obtaining these results, we hypothesized GRGDSP may reduce cell size and attenuate GLN-mediated protection by decreasing intracellular GLN concentration. As GRGDSP significantly reduced GLN-mediated cell size increases, it seemed likely that GRGDSP treatment would lead to reduced intracellular GLN content. Surprisingly, we found via LC-MS/MS that GRGDSP had no impact on intracellular GLN concentrations in basal or HS conditions (Fig. 3C). In our model of intestinal injury, increased intracellular GLN concentration does not appear to be required to initiate cell size changes and signaling via ERK to initiate cell protection pathways such as the HSP response. Thus, GLN appears to induce cell size changes via FN-Integrin signaling and potentially enhances HSP pathway activation in a manner that does not require increased intracellular GLN concentrations. If this osmosensing pathway via FN-Integrin interaction is specific to glutamine or if other cell swelling inducing amino acids funtion via the same osmosensing pathway will be investigated in future studies.

Since F-actin assembly plays an important role in inflammation and cell size regulation [12], [13] and is regulated via FN-Integrin signaling [14], one possible explanation is that GLN increases cell size via F-actin stabilization by FN-Integrin signaling, which activates the protective ERK and HSP response (Fig. 3D). When FN-Integrin interaction is interrupted by GRGDSP, the adhesion complex is not intact and cytoskeletal rearrangement may be inhibited, causing fragmented, disrupted, disorganized and collapsed actin (Fig. 3D). If the adhesion complex is not functioning baso-lateral actin may be disorganized, leading to cell adherence disruption. HS might cause disorganization of the cytoskeleton because FN expression is attenuated, leading to less FN-Integrin interaction and signaling, which causes apoptosis.

Next, we examined potential cell protective signaling pathways that could serve as downstream mediators of GLN-mediated FN-Integrin signaling. The ERK1/2 pathway is known to be activated by FN-Integrin signaling and can induce protective HSP expression following injury. This study shows for the first time that GLN activates ERK1/2 via the FN-Integrin signaling pathway. ERK1/2 assists in mediating transactivation of HSF-1 leading to increases in HSP70 expression, which is known to improve cell survival and to attenuate apoptosis after thermal injury (Fig. 2,3,4,5,6). This data may indicate that the recently demonstrated protective effect of fever in states such as human sepsis may depend not only on elevation of body temperature to initiate HSP expression (and other cell protective/immune response pathways), but also on sufficient GLN concentrations being available in the plasma or extracellular fluid at the time of injury. This hypothesis is consistent with data showing that deficient plasma GLN levels at ICU admission are associated with a significant increase in mortality [50].

Specific to the intestine, our data indicates under conditions of GLN depletion, intestinal epithelial cells are unable to mount an appropriate cytoprotective response to heat stress, resulting in apoptosis (Fig. 2). Further, GLN induces cell survival during physiological stress, potentially independent of changes in intracellular GLN concentration via induction of the FN-Integrin osmosensing pathway.

Herein, evidence is presented that GLN`s mechanism of cellular protection is mediated by two related pathways: 1) Via FN stabilization and 2) by the activation of the FN-Integrin cell volume sensing pathways in IEC-6 cells, leading to prevention of apoptosis. Fig. 7 proposes the pathway by which we hypothesize GLN mediates protection against intestinal epithelial cell injury via the FN-Integrin signaling pathway.

In conclusion, this study demonstrates that GLN stabilizes intestinal epithelial cell architecture by preventing FN degradation and actin destabilization after a mechanical injury, such as HS, via FN-integrin signaling. Mechanical injury interrupts cell-cell adhesion. ECM and tissue organization is important to keep the integrity of intestinal epithelial cells after gut injury. In this study, it could be shown that GLN depletion led to cell death because of loss of ECM and cytoskeleton architecture after HS. However, addition of GLN could improve epithelial tissue organization via FN-integrin-ERK signaling to prevent apoptosis after intestinal injury because cell-cell adhesion between neighbor cells was intact.

This GLN-mediated signaling may help explain the long known phenomenon of GLN mobilization from muscle and other stores to the plasma (and ultimately to the gut) following injury and illness. GLN mobilization and GLN-mediated cytoprotective signaling via the FN-Integrin pathway may be vital to intestinal cell survival and recovery from intestinal inflammation, critical illness, and other gut injuries**.** This data supports the use of GLN as a potential therapeutic agent to prevent and/or promote recovery from intestinal injury.

## Supporting Information

Figure S1 **ERK1/2 inhibitor UO126 attenuates GLN-mediated protection.** IEC-6 cells were treated with different concentrations of GLN (0–20 mM) with or without prior UO126 treatment. Cell survival was measured via MTS assay. All groups were normalized to their non-HS controls to account for differences in cell growth. Results are shown as mean±SEM (n = 3).
(TIF)

Figure S2 **GRGDSP affects GLN-mediated increases in HSP32 expression.** IEC-6 cells were treated with 0 mM or 10 mM GLN with or without 1 h prior GRGDSP or GRGESP treatment. Cells then underwent non-lethal HS (43°C). Western blot of HSP32 and ß-actin are shown. Results are ratioed to HS 0 mM GLN groups and represent means±SEM (n = 3).
(TIF)
