# Supplementary figures and images for: Fibronectin-Integrin Signaling Is Required for L-Glutamine’s Protection against Gut Injury
Source: PLoS One. 2012 Nov 20;7(11):e50185. doi: 10.1371/journal.pone.0050185 (PMC3502344; doi:10.1371/journal.pone.0050185)

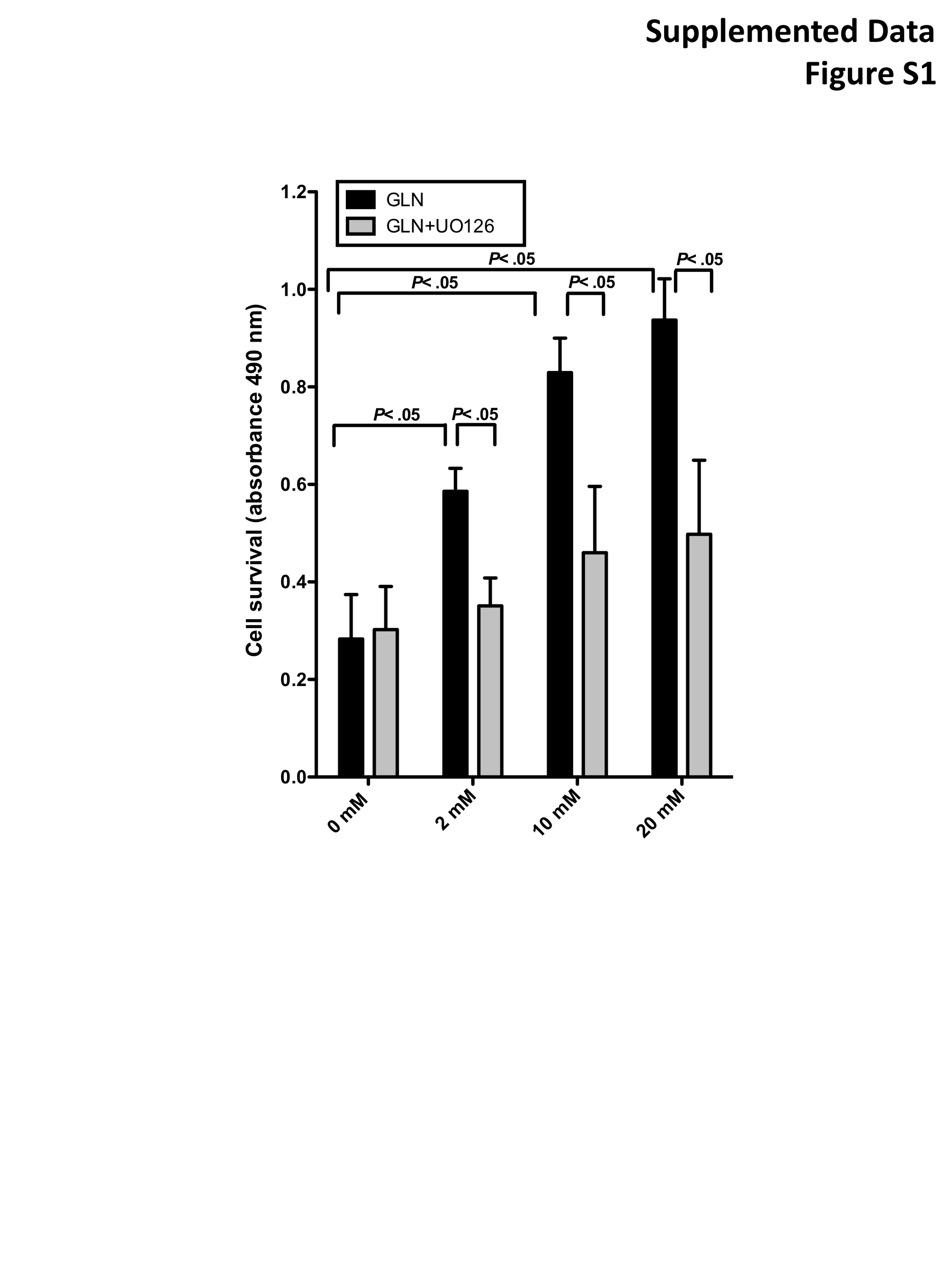

Supplement: Figure S1 — ERK1/2 inhibitor UO126 attenuates GLN-mediated protection. IEC-6 cells were treated with different concentrations of GLN (0–20 mM) with or without prior UO126 treatment. Cell survival was measured via MTS assay. All groups were normalized to their non-HS controls to account for differences in cell growth. Results are shown as mean±SEM (n = 3). (TIF) [file pone.0050185.s001.tif]

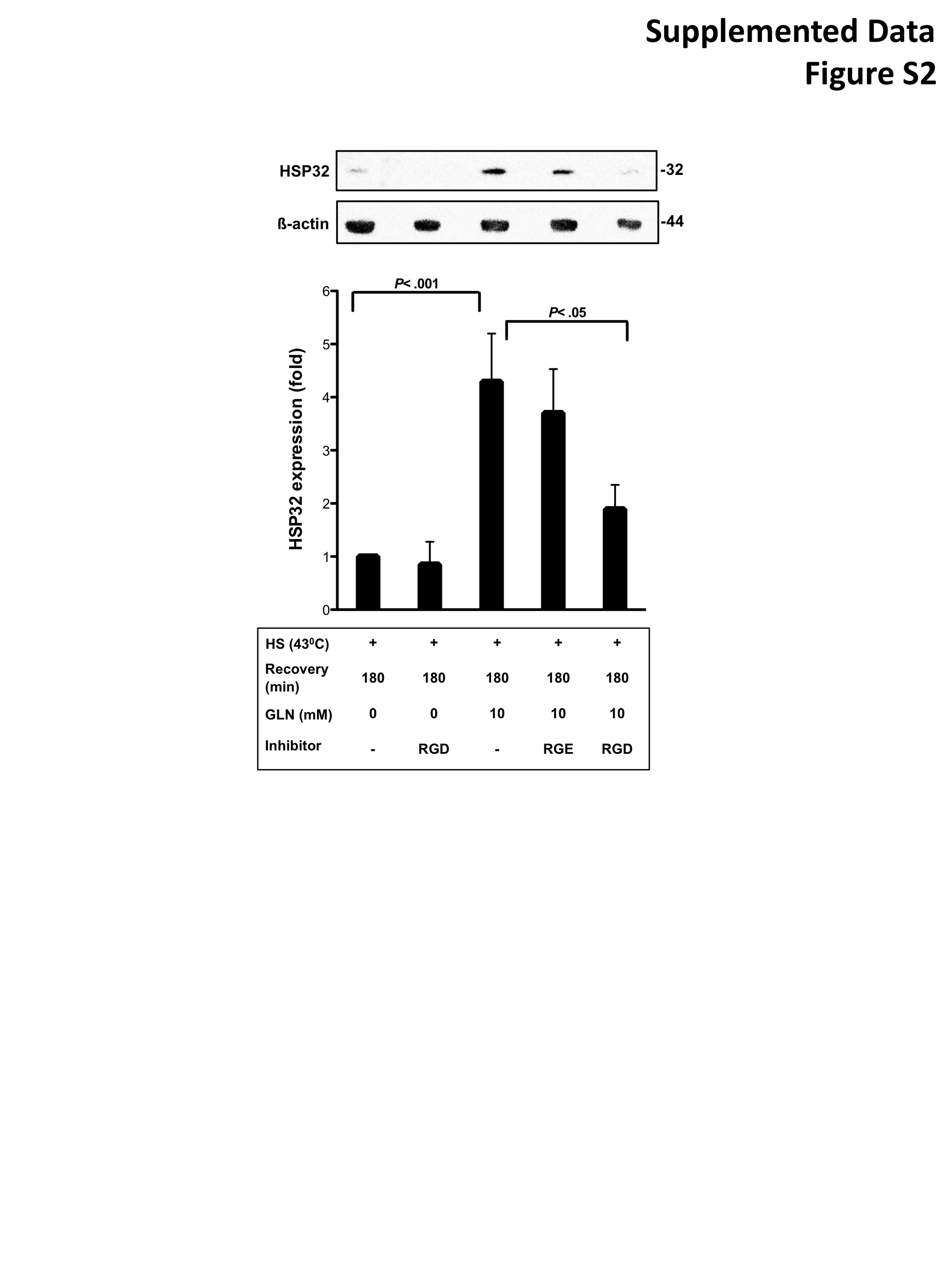

Supplement: Figure S2 — GRGDSP affects GLN-mediated increases in HSP32 expression. IEC-6 cells were treated with 0 mM or 10 mM GLN with or without 1 h prior GRGDSP or GRGESP treatment. Cells then underwent non-lethal HS (43°C). Western blot of HSP32 and ß-actin are shown. Results are ratioed to HS 0 mM GLN groups and represent means±SEM (n = 3). (TIF) [file pone.0050185.s002.tif]
